# Yale school of public health symposium on lifetime exposures and human health: the exposome; summary and future reflections

**DOI:** 10.1186/s40246-017-0128-0

**Published:** 2017-12-08

**Authors:** Caroline H. Johnson, Toby J. Athersuch, Gwen W. Collman, Suraj Dhungana, David F. Grant, Dean P. Jones, Chirag J. Patel, Vasilis Vasiliou

**Affiliations:** 1 Department of Environmental Health Sciences, Yale School of Public Health, New Haven, CT USA; 2 Department of Surgery and Cancer, Faculty of Medicine, Imperial College London, London, UK; 3 MRC-PHE Centre for Environment and Health, School of Public Health, Imperial College Norfolk Place, London, UK; 4 Division of Extramural Research and Training, National Institute of Environmental Health Sciences, National Institutes of Health, Morrisville, NC USA; 5 Waters Corporation, Metabolomics and Translational Research, Milford, MA USA; 6 Department of Pharmaceutical Sciences, University of Connecticut, Storrs, CT USA; 7 Department of Medicine, Emory University School of Medicine, Atlanta, GA USA; 8 Department of Biomedical Informatics, Harvard Medical School, Boston, MA USA

## Abstract

The exposome is defined as “the totality of environmental exposures encountered from birth to death” and was developed to address the need for comprehensive environmental exposure assessment to better understand disease etiology. Due to the complexity of the exposome, significant efforts have been made to develop technologies for longitudinal, internal and external exposure monitoring, and bioinformatics to integrate and analyze datasets generated. Our objectives were to bring together leaders in the field of exposomics, at a recent Symposium on “Lifetime Exposures and Human Health: The Exposome,” held at Yale School of Public Health. Our aim was to highlight the most recent technological advancements for measurement of the exposome, bioinformatics development, current limitations, and future needs in environmental health. In the discussions, an emphasis was placed on moving away from a one-chemical one-health outcome model toward a new paradigm of monitoring the totality of exposures that individuals may experience over their lifetime. This is critical to better understand the underlying biological impact on human health, particularly during windows of susceptibility. Recent advancements in metabolomics and bioinformatics are driving the field forward in biomonitoring and understanding the biological impact, and the technological and logistical challenges involved in the analyses were highlighted. In conclusion, further developments and support are needed for large-scale biomonitoring and management of big data, standardization for exposure and data analyses, bioinformatics tools for co-exposure or mixture analyses, and methods for data sharing.

## Background

Since the completion of the Human Genome Project in 2003, it has become apparent that most chronic diseases are attributable to both genetic and environmental influences; the environment is, however, the major contributor to the global disease burden [1–3]. In 2005, Christopher Wild put forth the concept of the exposome, which highlighted the need for comprehensive environmental exposure assessment tools to better delineate causality and disease [4]. The exposome, originally defined as “the totality of environmental exposures encountered from birth to death” [4], has moved from a concept to a reality and was recently redefined as the “cumulative measure of environmental influences and associated biological responses throughout the lifespan, including exposures from the environment, behavior, diet, and endogenous processes” [5]. To begin the epic task of attempting to analyze all exposures that an individual may encounter over their lifespan and connect those to biological impact, there have been significant technological advancements to enable the study of the exposome. Herein, the Department of Environmental Health Sciences at Yale School of Public Health (YSPH) brought together leaders in exposome technology advancements to discuss recent developments, limitations, and future needs, also with the intention to bring increased awareness to this growing and important field.

## Findings

The Symposium was initially opened by YSPH Dean Dr. Sten Vermund and National Institute of Environmental Health Sciences (NIEHS) Director Dr. Linda Birnbaum (by prerecorded video), who expressed the importance and value of the exposome in environmental health and epidemiology. The exposome is a highly interdisciplinary holistic approach that intersects environmental exposure monitoring with modern technologies such as genomics and metabolomics. It is a valuable science particularly important for understanding how environmental factors affect children’s health and later-life outcomes.

The Symposium discussed some current important considerations, developments, and future perspectives for exposome research which are discussed below.

## Moving away from a single exposure-disease approach

Environmental health research has traditionally focused on how a specific chemical, or class of chemicals, influences a specific health outcome. This single exposure-disease approach does not, however, represent the complexity of exposures encountered throughout life. Environmental exposures are complex, widespread, and play a role in human health and the development of disease. Researchers are now beginning to consider the totality of external and internal exposures across the life course, a concept called the exposome. Since the concept was developed, most efforts have focused on defining the exposome and challenges to its application into scientific research. Three studies in Europe—the Health and Environment-wide Associations based on Large population Surveys (HEALS), Human Early-Life Exposome (HELIX), and EXPoSOMICS projects—represent early coordinated efforts to advance exposome research [6, 7]. To further advance and promote implementation of the exposome in environmental health research, NIEHS convened a workshop in 2015 to review the state of the science and develop recommendations in key domains. Takeaways from each group included:

- Biomonitoring: Exploring cumulative exposure history requires a hybrid of traditional (targeted) and exposomic (untargeted) biomonitoring approaches and utilizing advantages of both methods [8].
- Biological response and impact: External and internal exposures interact to alter biological processes and trigger production of new chemical intermediates. Exposomic technologies can link exposures to these downstream effects [9].
- Epidemiology: The exposome is a complement to environmental epidemiology. Untargeted analyses can generate findings that need to be investigated using hypothesis-driven approaches central to epidemiology. Merging data across cohorts with different life stages enables characterization of the exposome across the life course [10].
- Data science: Exposomic approaches generate extensive data to be stored, managed, analyzed, integrated, and shared. Development of community-based data standards and ontologies is critical [3].

While specific challenges exist in each domain, there are cross-cutting themes and needs, central to implementing the exposome into scientific research. Infrastructure support is needed for large-scale untargeted biomonitoring and managing big data. Advancement and standardization of technologies and methods for exposomic analyses as well as data analyses, integration, and sharing are another area requiring considerable support. Training environmental health scientists in big data and use of exposomic approaches in human health research is also critical. Promotion of the concept in research areas that can benefit from the exposome approach, such as epidemiology, can accelerate its implementation.

The NIEHS Children’s Health Exposure Analysis Resource (CHEAR) Program addresses many of these challenges. CHEAR laboratory services span the breadth of the exposome and offer targeted and untargeted analyses. The CHEAR Data Center serves as a data repository, providing statistical analyses, data integration, and interpretation services. This data center is also developing community-based data standards and ontologies. NIEHS supports a hybrid approach to investigating the exposome, which depends upon interactive workshops, established resources (e.g., CHEAR), and new research initiatives. In these efforts, NIEHS interacts with other institutes within the National Institutes of Health, as well as with other federal agencies and international consortia as mentioned above. By taking this comprehensive approach, the exposome can be implemented into environmental health research.

## Inter- and intraindividual variability and analytical advancements

Many burdensome diseases are complex and result from a combination of hereditary and environmental factors. For example, the heritability, or the phenotypic variation in the population attributable to inherited factors is on average 50%, leaving the other 50% of phenotypic variation to specific influences of the environment [11]. Additional analytic tools and data are needed to discover exposures to explain missing phenotypic variation in the population. In contrast, genomic investigations have accelerated the pace for discovery of inherited factors in disease, and the same advances should be applied to discover the influences of exposures in disease [3]. For exposome studies to deliver the promised benefits and causal understanding we seek, and to inform public health policy and chemical risk assessment, it is important to understand metabolic (and other) networks in a holistic manner to link toxic exposures, responses, and disease endpoints. However, modeling such networks may be confounded by uncertainty in both the interactions that occur and the variability of network components; some components of urinary and blood metabolomes (pool of low molecular weight compounds in the sample) exhibit high variability, whereas, others appear to be tightly regulated. Contributions of genetic and environmental factors to metabolic phenotypes have been identified, alongside differences in stability and resilience of components of the metabolomes over long periods in individuals [12, 13].

In addition, inter- and intraindividual variability in specific subpopulations or strata that are of importance to exposome studies should be characterized, including those in critical periods of life (including in utero, early life, and old age) where susceptibility to adverse consequences of exposure may be increased. Such subpopulations exist in the European Union FP7-funded HELIX project. This project is focused on studying the early life exposome by combining six mother-child cohorts in Europe: BiB (Born in Bradford, UK), EDEN (Étude des Déterminants pré-et postnatals du développement et de la santé de l’Enfant, France), INMA (INfancia y Medio Ambiente, Spain), KANC (Kaunus Cohort, Lithuania), MoBa (Norwegian Mother and Child Cohort Study, Norway), and the Rhea Mother-Child Cohort Study (Rhea, Greece) [6]. Within these cohorts, a total of 1200 mother-child pairs were selected for exposome characterization using a multitude of analytical approaches, including both internal and external measures. To assess variability in one of the measured parameters, the metabolome, a panel study within the INMA component of the subcohort was conducted to investigate the temporal variability of metabolite concentrations in children’s urine over a week, relative to the interindividual variation. Variations attributable to experimental and biological factors (analytics, cohort, etc.) were assessed, giving a better understanding of the sources of variability in these metabolic phenotyping data [14]. The study showed that one of the sources of variation was diurnal and resulted in the recommendation that a pooled sample should be used made from urine samples collected at different times of the day, or a 24-h urine collection for exposome analysis. There was also interindividual variability in metabolites between males and females (citric acid) and in dietary metabolites such as *N*-methylnicotinic acid, which is derived from coffee, soda drinks, and chocolate.

An approach called “exposome-wide association studies” or equivalently “environment-wide association studies” (EWAS) is another methodology to drive discovery of new exposures in disease and the missing phenotypic variation in the population (e.g., [15–18]). EWAS offers a few advantages, including explicitly mitigating false positive findings and assessing the entire database of potential environmental correlates systematically to avoid a fragmented literature of associations [19–21]. While far from causal (and observational), the associations that emerge are those that can be prioritized to investigate in biological experiments. For example, Patel and colleagues, after using an EWAS-like approach to find exposure factors putatively correlated with telomere length, investigated how the exposure factors potentially influence changes in gene expression using publicly available data from the *Gene Expression Omnibus* [22]. Exposures must influence changes in biological function if causal, and gene expression investigations are among the important approaches to decipher causal routes to disease.

## Recent technological advancements in metabolomics for exposomics

Exposome studies ultimately promise to help identify causal pathways that link environmental exposures to disease endpoints, by combining a range of state-of-the-art technologies that probe external and internal exposures, and concomitant responses. Central to the task of implementing human exposome studies is to characterize the metabolome, a phenotypic measure that encodes a wealth of information relating to genes, environmental exposures, and their various interactions [23–25]. Accordingly, high-resolution platforms such as ultraperformance liquid chromatography-mass spectrometry (UPLC-MS) and ^1^H nuclear magnetic resonance (NMR) spectroscopy for generating metabolic phenotypic data are now commonly included in molecular epidemiological studies of chronic disease [26, 27]. For exposomics, it is essential that these platforms can (1) capture the diversity of the chemical space, (2) assign chemical identify to the associated mass spectral features, and (3) accurately quantify them. Both mass spectrometry (MS) and NMR spectroscopy have been used for analyses of the exposome; however, MS was a predominant focus of this Symposium which highlighted advancements in hardware and software for large-scale exposome analysis.

High-resolution metabolomics (HRM) of plasma has the potential to be a central platform for affordable, high-throughput, biomonitoring of environmental chemical exposures [28, 29]. The platform takes advantage of the ultra-high mass resolution, mass accuracy, sensitivity, and improved scan speed of modern Fourier-transform (FT) mass spectrometers. Improved data extraction methods support routine measurement of more than 20,000 mass spectral features in microliter volumes of plasma and other human samples, and advanced software packages support both knowledge-driven and data-driven approaches for interpretation of these data. These developments, listed below in more detail, create exciting new opportunities for large-scale, systematic exposome research.

### Fourier-transform mass spectrometry

Comisarow and Marshall [30] showed that FT of signals from the free induction decay (FID) of ions responding to an oscillating electric field that is orthogonal to a fixed ion cyclotron resonance (ICR) magnetic field could be used to measure the mass-to-charge ratio (*m*/*z*) of an ionized metabolite. Application of FT-ICR MS to crude oil showed that 17,000 ions could be detected [31]. These instruments showed linear response characteristics for quantification over at least five orders of magnitude [32], but were limited for high-throughput analyses because of the slow scan speed required, and thus run times needed of 10 minutes or longer. Introduction of a new FT instrument, termed an Orbitrap [33], supported faster scan speeds, and scan speed has been further improved with a high-field version. Importantly, unlike time-of-flight instruments, the mass resolution and mass accuracy are preserved at low ion intensities, and this allows quantification of chemicals over nearly eight orders of magnitude [34]. This extensive dynamic range is an important capability for high-throughput exposome research because environmental chemicals are frequently encountered three-to-five orders of magnitude lower abundance than endogenous metabolites [35]. Early results from gas chromatography (GC)-Orbitrap analyses provide optimism for expansion of high-throughput biomonitoring to include more non-polar, hydrophobic environmental chemicals. Hundreds of environmental chemicals in different pesticide groups have been detected in single analyses of human samples (D.I. Walker, unpublished). Thus, a combination of liquid chromatography (LC) and GC analyses could provide a standardized central platform for longitudinal studies to initiate a human exposome project. Results are already available to show that analyses can be performed on samples stored for decades [36]. Therefore, rapid progress can be made by analysis of existing samples for which health outcome data are available. These high-throughput methods could provide a central resource on which to build a more extensive exposome research capability [28, 29].

### Large-scale, systematic exposome research

Humans are thought to experience more than a million exposures in a lifetime [37], so detailed understanding of life-stage-specific effects represent an enormous challenge. Using the Human Genome Project as a reference, one can focus on several criteria needed for exposome research explained below. These criteria have already been established for HRM, which further supports the use of HRM for exposome research.

First, assays need to be simple enough that they can be easily performed in multiple facilities; samples can now be processed using just a single extraction and centrifugation prior to analysis [38]. Second, assays need to be high-throughput and automated with extensive coverage; the extraction and injection workflow for LC-MS can be fully automated, and run-times can be as short as 2.5 min (to detect more than 20,000 mass spectral features in batches of samples) [29]. Third, the assays need to be reproducible; analyzing each sample in triplicate, one can verify the reproducibility of an individual signal in an individual sample; however, a second biological sample set is always necessary when available. Fourth, standards are required; reference standardization [34] has been developed to support creation of cumulative data libraries; a pooled reference sample is calibrated against a National Institute of Standards and Technology (NIST) Standard Reference Material (e.g., SRM1950 containing approximately 100 metabolites) and concurrently analyzed with each batch of 20 samples. This analytic structure allows estimation of the absolute concentration of any known compound that is measured in the NIST standard. There are numerous standards available for different environmental chemical classes such as pesticides, organic contaminants from cigarettes, chemicals in drinking water, drugs, and their online spectral library are for approximately 267,376 chemical compounds. However, we know from a recent expansion to the METLIN metabolite database that the number of endogenous and environmental chemical compounds is at least 1 million, so some standards are still needed for complete coverage [39]. Finally, fifth, assays need to be affordable; the use of a dual chromatography [40], and dual ionization approach [41], provides a very broad spectrum of chemicals that can be analyzed in triplicate for about $100/sample [28], with possibilities to lower this to below $10/sample [36].

### Tools for data extraction and analysis

Extraction of ultra-high resolution mass spectral data can be effectively obtained with software language tools such as apLCMS [42] and XCMS [43]. A wrapper function in xMSanalyzer [44] allows re-extraction of the same data with different parameter settings to improve capture of signals with different characteristics, and also provides quality control, batch normalization, and other capabilities. xMSannotator [45] provides an automated computational framework using existing databases (ChemSpider, KEGG, HMDB, T3DB, LipidMaps) and a multistage clustering algorithm to assign confidence scores for chemical identity by evaluating intensity profiles, retention time characteristics, mass defect, isotope patterns, adduct patterns, and correlations with matches to other metabolites in known metabolic pathways. The resulting annotated data are compatible with any of the many pathway analysis tools, e.g., KEGG [46], MetaboAnalyst [47] and MetaCore [Thomson Reuters (https://portal.genego.com/)]. To complement these knowledge-based tools, which require knowledge of chemical identities prior to pathway analysis, *mummichog* is a set of computational algorithms to predict metabolic pathway effects directly from spectral feature tables without prior identification of metabolites [48].

### Chemical identification

One of the major issues for metabolomics in studies of the exposome has been chemical/structural identification. Less than half of the ions detected by mass spectrometry are associated with known chemicals and essentially the rest are the “dark matter of the exposome.” Of critical importance for exposome research, about half of the mass spectral features associated with disease are currently unidentified [37]. It was recognized early on that the structural identification of detected metabolites might be problematic [49]. However, this was considered a temporary problem, easily solved by constructing databases containing all known mammalian metabolites. At the time, the mammalian metabolome was estimated to contain ≤ 5000 compounds, so the assertion that a complete metabolite database would be readily available seemed reasonable. Thanks to the diligent work of many individuals, there are now several excellent and freely available metabolite databases (METLIN, HMDB, KEGG, HumanCyc, and others) that contain these 5000 compounds, and many more [50–53]. Unfortunately, despite the availability of databases, high-throughput instrumentation, ophisticated statistical, and cheminformatics tools, the “structure identification” problem has not been solved. What has replaced the initial expectation of identifying the structures of hundreds or thousands of metabolites is the disappointing realization that most discovery metabolomics studies report the chemical structure of fewer than 100 compounds, and very often less than 50 [54]. Thus, the most significant barrier to progress and a seemingly intractable limitation of the field of metabolomics is the inability to identify the structures of most detected compounds.

A novel approach to help overcome this problem is the development of algorithms that predict physical/chemical properties of compounds contained in chemical databases. The properties chosen are those that can be experimentally measured for any unknown compound by LC-MS. These include retention indices, precursor ion survival curves, collision-induced dissociation fragmentation spectra, biological relevance, and collisional cross-sectional area. Compounds in databases (for example PubChem) whose predicted properties most closely match experimentally measured properties are returned as the most likely candidates for the unknown peak. This system is currently being validated in an in vivo model of acute trauma and could be of value for exposome studies. In addition, a multilevel framework to include unidentified signals in cumulative databases has been proposed in which ion characteristics are defined in multivector space, which will aid in overcoming this problem [37].

Orthogonal technologies in MS can also separate and capture the chemical complexity of the samples, aiding structural identification. LC is a proven technology that is orthogonal to MS, and it can be adequately optimized to target the chemical space of interest, for example, hydrophilic interaction liquid chromatography is appropriate for hydrophilic molecules while a reversed-phase method provides optimal separation for lipophilic molecules. The correct chromatography allows separation of isomers and removes isobaric interference in a mass spectrometer and makes identification of unknowns easier. Similar to LC, ion mobility, a gas phase separation of ions based on their mobility, has been shown to be very useful in separating and capturing the components of a complex sample and providing with clean mass spectra needed for identification [55]. It has been recognized that appropriate fractionation of samples at the sample preparation step as well as on-line separations (chromatography and ion mobility) all assist in capturing the chemical complexity of a matrix, such as blood, by decoupling the overlapping interferences. Accurate quantification is the next logical step following detection and identification of exposure-associated molecular species. Molecular species originating from food and drugs fall in the same concentration regime as endogenous metabolites, while the blood concentration levels of environmental pollutants are 1000 times lower. Modern mass spectrometers have a dynamic range of six orders of magnitude, therefore assessment and validation of the dynamic range of the analytical assay used to measure these molecular species are critical [56]. Dynamic range of an analytical method is defined as the concentration range over which a signal response is linear to the concentration. It is important that the dynamic range in exposure analysis be defined in the context of a molecule, especially in MS-based analysis where the ionization of molecular ions can be vastly different. Furthermore, interference in the samples can easily result in ion suppression or enhancement, which can result in inaccurate quantification. Use of hyphenated technologies such as LC-MS is even more important in quantification since chromatography can separate out the interfering ions and alleviate ion suppression or enhancement challenges.

### Integrated omics methodologies

An important need is to link external exposures to internal body burden, and internal body burden to biologic responses and disease outcomes. A recent example of this analysis continuum was presented in an HRM study of occupational exposure to trichloroethylene (TCE) [57]. This study showed that TCE exposure was associated with known TCE metabolites in the blood and urine and that other halogenated compounds were correlated both with TCE metabolites and disease biomarkers. Thus, the results showed the use of HRM to connect external exposures, internal body burden, biologic response, and biomarkers of disease outcome. These analyses used a new R script, xMWAS [https://sourceforge.net/projects/xmwas/ ] [58], which advances capabilities to integrate HRM data with any other omics data, as already shown for transcriptome x metabolome [59], microbiome x metabolome [60], cytokine x metabolome [61], and suitable for genome, epigenome, proteome, and other integrated omics analyses that will be essential for mechanistic understanding of the exposome.

## Current limitations in exposomics

### Mixtures of exposure profiles

One major limitation of approaching exposure-disease associations is the number, diversity, and mixture of environmental exposure profiles at the individual level; such an array of exposures is a challenge for both measurement science and in efforts to identify components or combinations that are associated with adverse outcomes. Several publications have shown how perturbations in metabolic networks (and others such as those of gene regulation and protein-protein interactions) can help describe complex phenomena such as multimorbidity; nodes in the underlying biochemical networks connect disease phenotypes [62, 63]. It may, therefore, be useful to consider a set of these network perturbations as endpoint proxies when identifying the effects of exposure. Similarly, the conceptual framework for defining adverse outcome pathways (AOPs), i.e., making causal links from molecular initiating events (MIEs) to subsequent key events (KEs), may help reduce the complexity through identifying common MIEs for multiple environmental stressors [64].

### False-positives

With big data come big analytical challenges. EWASs are observational studies and prone to biases such as confounding and reverse causality (e.g., disease coming before the exposure) [65]. While there are analytic and epidemiological study designs that attempt to mitigate these biases, they have yet to be harnessed in high-throughput exposure investigations. Yet another issue includes multiple significant associations (low *p* values) with small effect sizes. A recent “prescription-wide association study” on time-to-cancer in a large and comprehensive Swedish database [66] found that when examining > 500 drugs prescribed, almost a quarter of them were associated with cancer with small effect. Furthermore, these effects changed as a function of analytic method chosen (e.g., case-crossover vs. Cox proportional hazard), leaving an investigator with many potential false positives to sift through. This may be the norm in exposome-wide analytics, where correlations may be small but seemingly correlated with everything else and it will be a challenge to ascertain causality [67, 68].

## Future perspectives

With respect to the future, the thirst for metabolic phenotype data, to complement other available omics, personal monitoring, and activity data, is only likely to increase, as the concept of the human exposome is developed and deployed around the world. As mentioned previously, methods that can integrate metabolomic data with other system-level data can provide valuable insight for exposomics and provide a way to link these datasets together in a meaningful way. It is therefore timely that several national and international initiatives have been started to provide high-throughput, multiplatform analyses with the capacity to serve the wider scientific community. These initiatives will benefit from a renewed commitment to analytical and data standards harmonization, and opportunities for wider access through open data agreements. If the recent efforts to make large, well-curated, EWAS datasets available alongside tools for their interrogation (e.g., Patel et al. [69]) can be mirrored for MWAS, then progress in exposome studies is likely to move forward at pace.

While recent advances in MS have been enabling, we still need to take advantage of the orthogonally complementing technologies to separate and capture the chemical complexity of the samples. In addition, standardization needs to be addressed, as more exposome studies are developed, reproducibility across sample cohorts will inevitably become an issue. As biobanks are being amassed, there is no current standardization for sample collection, thus data generated will be biased to collection procedure and quality of sample during long-term storage. Currently, data repositories exist for metabolomics and genomics but are missing important additional detailed information such as the type of sample collection tube, time between collection and freezing, number of freeze-thaw cycles, and analytical platform used (vendor/model). Thus, these important points need to be considered for widespread integration of the exposome.

## Conclusions

In a new era of high-throughput environmental exposure assessment, there is an urgent need for new analytic approaches to drive discovery of new exposures associated with disease and phenotype [70]. Herein, the Symposium provided increased awareness of the utility of the exposome in environmental health research, as well as recent updates on technological innovation and challenges that still need to be overcome to enable the study of the exposome, particularly in metabolomics. Despite these challenges, the exposome paradigm may bear fruit in enhancing discoveries to identify exogenous factors that have up to now been elusive in disease risk. Moving forward, new technologies to measure environmental exposures in large populations in a cost-effective manner are needed, along with harnessing sound epidemiological designs to extract potential signals from noise, with data deposited in the public domain for investigators of all ilk to analyze results.
